# Osteoblast Role in Rheumatic Diseases

**DOI:** 10.3390/ijms18061272

**Published:** 2017-06-15

**Authors:** Addolorata Corrado, Nicola Maruotti, Francesco Paolo Cantatore

**Affiliations:** Rheumatology Clinic, Department of Medical and Surgical Sciences, University of Foggia Medical School, 71122 Foggia, Italy; ada.corrado@unifg.it (A.C.); nicomaruotti@libero.it (N.M.)

**Keywords:** arthritis, osteoarthritis, osteoblast, osteoporosis, spondyloarthritis

## Abstract

Alterations in osteoblast growth, differentiation and activity play a role in the pathogenesis of several rheumatic diseases, such as rheumatoid arthritis, spondyloarthritides, osteoarthritis, and osteoporosis. In fact, in these rheumatic diseases, abnormal activity of Wnt signaling, receptor activator of nuclear factor-κB (RANK)-RANK ligand (RANKL)-osteoprotegerin (OPG) signaling, bone morphogenetic proteins (BMPs) pathway and other mechanisms have been described in osteoblasts. This review article is focused on current knowledge on the role of osteoblast dysregulation occurring in rheumatic diseases.

## 1. Introduction

Osteoblasts play a role in the pathogenesis of several rheumatic diseases. Alterations in osteoblast growth, differentiation and activity have been described in chronic inflammatory diseases, such as rheumatoid arthritis (RA) and spondyloarthritides (SpA); in degenerative joint diseases, such as osteoarthritis (OA); and bone diseases, such as osteoporosis. This review article is focused on analysis of the current knowledge on the role of osteoblast dysregulation occurring in rheumatic diseases.

## 2. Osteoblast Physiology

Osteoblasts are mononuclear specialized cells originated from pluripotent mesenchymal stem cells differentiation via activation of specific signaling transcription pathways, such as osterix (Osx), Runt-related transcription factor 2 (Runx-2), and Wnt pathway [1].

Osteoblasts play a key role in the regulation of bone metabolism. In fact, they are involved in the production of bone matrix constituents, such as type I collagen. Subsequently, osteoblasts influence calcium and phosphate local concentrations and induce the following bone matrix mineralization [2,3]. Moreover, osteoblasts produce sialoprotein, osteopontin and osteocalcin, which have been associated with the mineralized matrix in vivo [1,4].

On the other hand, osteoblasts are involved in osteoclast regulation by expressing on their membrane or releasing as soluble factor, receptor activator of nuclear factor (NF)-κB ligand (RANKL).

The interaction between RANKL and receptor activator of nuclear factor-κB (RANK), a type I transmembrane receptor expressed on osteoclast precursors, is responsible for inducing osteoclast precursor differentiation into osteoclast. The binding of RANKL to RANK leads to the trimerization of RANK and the activation of tumor necrosis factor (TNF) receptor-associated factor 6 (TRAF6). Subsequently, TRAF6 induces the activation of NF-κB and mitogen-activated protein kinases (MAPKs), such as p38 and Jun N-terminal kinase (JNK), which are, in turn, involved in the activation of transcription factors such as c-Fos, c-Src, and microphtalmia transcription factor (MITF) [5,6,7]. Furthermore, osteoblasts may modulate osteoclastogenesis through the production of osteoprotegerin (OPG). OPG is a soluble decoy receptor for RANKL, which is responsible for a competitive inhibition of RANK/RANKL link, thus avoiding RANK activation [8].

Numerous physiological osteoblast aspects, such as osteoblastogenesis and OPG expression are regulated by the Wnt/β-catenin signaling pathway [9,10,11]. Wnt binding to its coreceptor complex located at the cell surface, which is constituted by the low-density lipoprotein receptor-related proteins 5 (LRP-5) or 6, and a member of the frizzled (Fz) family of proteins, induces Frizzled activation and the recruitment of cytosolic Disheveled (Dvl) proteins [12,13]. Cytosolic β-catenin is phosphorylated by glycogen synthase kinase 3 (GSK3) and casein kinase 1 (CK1), which form a complex together with Axin and the tumor suppressor adenomatous polyposis coli (APC) [14]. β-catenin phosphorilation favors its subsequent degradation via ubiquination. Dvl inhibits β-catenin degradation and allows its cytoplasmic accumulation and nuclear translocation. In the nucleus, β-catenin is involved in activating the expression of several target genes [11,15,16,17]. Wnt signaling is inhibited by secreted Fz-related protein family (sFRP) and Wnt inhibitory factor-1 (WIF-1) [18], which block the interaction of Wnt with its receptor Fz. Moreover, LRP5/6 is blocked by proteins of the Dickoppf (DKK) family and by sclerostin, a glycoprotein that is mainly secreted by osteocytes [19].

Osteoclastogenesis regulation involves also several other molecules produced by osteoblasts, such as interleukin-1 (IL-1), TNF-α, and macrophage-colony stimulating factor (M-CSF). IL-1 in the presence of adequate levels of RANKL is a potential regulator of osteoclastogenesis. In osteoclast precursor and marrow stromal cells, IL-1 may activate p38 MAP-kinase, which plays a role in TNF-α-mediated osteoclastogenesis [20]. TNF-α stimulates RANKL production both directly by producing factors such as TRAF6, RANK, and NF-κB, which are responsible for the activation of osteoclast precursor cells in the early phase of osteoclastogenesis, and indirectly by stimulating osteoclastogenesis-supporting mesenchymal cells [21,22,23,24]. TNF receptor type I (TNFRI) and TNF receptor type II (TNFRII) are involved in TNF-α biological activity. Nevertheless, only TNFRI seems to be involved in RANKL-induced osteoclastogenesis, as suggested by the suppression of RANKL-induced osteoclastogenesis after addition of neutralizing anti-TNFRI, but not anti-TNFRII antibodies [25,26]. M-CSF has a role in recruiting osteoclasts, as observed in mice with mutation in the coding region of the M-CSF, which were affected by osteoclast-deficient osteopetrosis [27]. M-CSF may activate c-fms, a tyrosine kinase receptor that in turn promotes ERK1/2 and PI3-K/AKT activation, two signaling pathways involved in osteoclast differentiation [28]. Moreover, M-CSF induces osteoclast differentiation via inducing the expression of RANK on pre-osteoclasts surface [29].

A further control in the regulation of RANKL and OPG expression in osteoblasts is played by vitamin D, calcitonin, estrogen, parathormon (PTH), serotonin and leptin [1]. Osteoblast growth and differentiation is also regulated by bone morphogenetic proteins (BMPs), such as BMP-2, BMP4 and BMP-7 which are multi-functional growth factors that belong to the transforming growth factor-β (TGF-β) superfamily [1].

## 3. Osteoblast Role in RA

RA is characterized by an increase in bone resorption and impaired bone formation. This imbalanced bone remodeling has been observed both in the subchondral and periarticular bone of joints, where it results in erosions and periarticular osteopenia, and in the axial and appendicular skeleton, where it results in a generalized bone loss.

### 3.1. Focal Articular Bone Loss

Even if other cells, such as osteoclasts, macrophages, and synovial fibroblasts, are mainly involved in these manifestations, osteoblasts probably play a role in the pathogenesis of bone erosions in RA. By using hystomorphometric analysis in a murine model of RA, Walsh et al. [30] have observed that the rate of bone formation in bone surfaces adjacent to inflammation were similar to those observed in non-arthritic bone, suggesting that osteoblasts are not able to balance osteoclast resorption. Moreover, within arthritic bone, the extension of mineralization in the newly formed bone was reduced in areas adjacent to inflammation compared with bone surfaces adjacent to normal marrow, suggesting that inflammation modifies osteoblast physiology.

In arthritic joints, the bone microenvironment is exposed to reduced pH and hypoxia. By considering that acidosis and hypoxia down-regulate osteoblast production of alkaline phosphatase and inhibit mineralization in murine osteoblast cultures [31,32], it is conceivable that osteoblasts exposed to the same conditions in the arthritic joint bone microenvironment, may be characterized by reduced activity. In particular, hypoxia inhibits Wnt signaling in osteoblast-like cells, both directly, by inhibiting transcriptional activity via blocking β-catenin [33], and indirectly, by the up-regulation of DKK-1 [34].

TNF-α and Wnt signaling pathway are probably involved in osteoblast maturation and functions at sites of bone erosions. TNF-α is probably involved in dysregulation of Wnt signaling via inducing DKK-1 over-expression in synovial fibroblast cells [35]. In RA murine models, high levels of TNF-α have been associated with increased levels of DKK-1, and the treatment with DKK-1 antibodies has shown protective effects against bone erosions, suggesting that this is related to the inhibition of the negative regulation of osteoblast maturation and activity [35]. Sclerostin, an inhibitor of the Wnt signaling, inhibits osteoblast differentiation. By using human TNFα transgenic (hTNFtg) mice affected by RA-like disease, Wehmeyer et al. [36] found that the lack of sclerostin or its antibody-mediated inhibition were responsible for an enhanced pannus formation and joint destruction.

TNF-α administration in vitro inhibits osteoblast-related matrix mineralization [37]. Moreover, TNF-α is responsible for a reduction in collagen type I synthesis, in alkaline phosphatase activity, and in osteocalcin expression in vitro [38,39,40]. An anti-osteoblastic effect has been also described for IL-6, which is most likely mediated by its negative interaction with Wnt signaling pathway [41].

The exposition of osteoblast-like cells to sera of RA patients treated with TNF-α inhibitors has been associated to a reduced synthesis of IL-6, a cytokine directly responsible for arthritis-related bone loss [42]. The same results were obtained also by direct administration of TNF-α inhibitors to osteoblast cultures [43].

The osteoblast expression of pro-inflammatory cytokines, such as IL-1, IL-6, and IL-8, is also favored by adiponectin. Adiponectin induces ostoblast production of mediators that drive synovitis and joint destruction such as matrix metallo-proteinase-1 (MMP-1) and MMP-13, and vascular endothelial growth factor (VEGF) [44,45]. Recently, Chen et al. [46] have observed that the pro-inflammatory cytokine Cysteine-rich 61 (Cyr61 or CCN1), a secreted protein from the CCN family, induces VEGF expression in osteoblasts and increases endothelial progenitor cells angiogenesis in RA. Adiponectin is also involved in RA bone remodeling by inhibiting Osx and inducing OPG expression in osteoblasts [44].

Moreover, overexpression of miR-221 has been seen to be involved in inhibition of calvarial osteoblast differentiation and mineralization in vitro. These results suggest that miRNAs derived from inflamed synovial tissues play a key role at erosion sites in signaling pathways that influence bone loss and potentially also compensatory bone formation [47].

### 3.2. Generalized Bone Loss

Osteoporosis is more frequent in RA patients than in the healthy population [48]. In RA patients also affected by osteoporosis, increased levels of RANKL and decreased levels of OPG have been demonstrated [49]. In active RA lower serum osteocalcin, which is a marker of bone formation, and higher crosslinked *N*-telopeptidases of type 1 collagen (NTX) and deoxypyridinoline (DPD), which are markers of bone resorption, have been found compared to controls and patients with inactive rheumatoid arthritis [50]. Among the main factors involved in RA-related osteoporosis are inflammatory cytokines, glucocorticoid therapy, reduced physical activity, low calcium intake, and poor nutrition associated with enhanced basal energy expenditure [48]. In RA, inflammatory cytokines, such as TNF-α, IL-1, IL-6, and IL-17 are involved in the activation of osteoclastogenesis via inducing over-expression of RANKL [51].

Even if further studies are needed to better understand the role of osteoblasts in generalized bone loss in osteoporosis, histomorphometric studies have indicated that, in RA patients who have never been treated with glucocorticoids, osteoporosis seems to be due to a decrease in bone formation rather than to an increase in bone resorption [52,53], suggesting that a reduction of osteoblast activity is involved in generalized bone loss in RA.

## 4. Osteoblast Role in SpA

Osteoporosis has been reported as a common complication in SpA patients. Reduced physical activity due to joint pain and worsen back mobility, a more frequent incidence of inflammatory bowel diseases with the subsequent intestinal malabsorption, glucocorticoid treatment, and increased levels of inflammatory cytokines, such as TNF-α, IL-1, IL-6, and IL-17, play a key role in SpA-related osteoporosis [54].

Despite the frequent evidence of osteoporosis, sites of joint and vertebral inflammation in SpA patients are characterized by increased local bone formation [55,56]. Entheses, the attachment sites of tendon, ligament, joint capsule, fascia or muscle to bones, are considered as the primary target tissue for inflammation in these diseases.

TNF-α is involved in the initiation and regulation of the inflammatory response, by inducing inflammatory cytokines such as IL-1 and IL-6, by recruiting immune and inflammatory cells and by inducing the expression of adhesion molecules as suggested by high levels of TNF-α observed in patients with ankylosing spondylitis (AS) and psoriatic arthritis (PsA) [57,58]. TNF-α is responsible for inducing osteoclasts differentiation and function, while concomitantly is involved in the osteoblastogenesis inhibition [56]. Other inflammatory cytokines, such as IL-17 and IL-23 play a central role in abnormal bone formation in AS [59]. IL-23 in enthesial tissues is responsible for inducing a subset of enthesial resident T cells, identified as RORγT+CD3+CD4−CD8−, to produce IL-17 and IL-22 [60]. IL-22 is involved in inducing the differentiation of osteoblasts at the enthesial sites from precursors cells [59].

Numerous evidences suggest a role for an abnormal activation of BMPs pathway and Wnt signaling in excessive periosteal and syndesmophyte bone formation in SpA [61,62,63,64].

Low levels of inhibitors of Wnt signaling, such as DKK-1 and sclerostin are involved in inducing bone formation in SpA. In AS patients high DKK-1 levels were correlated to a lower syndesmophyte formation in the spine than patients with low DKK-1 levels, suggesting that low DKK-1 levels play a key role in the pathological bone formation in AS [63]. Recently, Kragstrup et al. [65] have suggested a role for IL-20 and IL-24 in decreasing the production of DKK-1 by SpA fibroblast-like synovial cells and inducing mineralization in human osteoblasts. Moreover, Lee et al. [66] have found higher IL-32γ levels in synovial tissues and fluids of AS patients than in patients with RA or OA. They also demonstrated that IL-32γ was involved in inducing osteoblastogenesis and reducing the expression of DKK-1.

Similarly, low sclerostin levels have been found in sera and periarticular bone from AS patients, with an inverse correlation between radiographic progression of bone formation and sclerostin levels [62].

## 5. Osteoblast Role in OA

Several factors involved in bone metabolism are dysregulated in OA. Alterations in the expression of OPG and RANKL have been found in OA osteoblasts [67,68]. Low levels of R-spondin-2 (Rspo-2), a Wnt/β-catenin signaling pathway agonist, and high level of sclerostin, have been found in primary human osteoarthritis osteoblast cultures [69,70].

Moreover, an over-expression of RUNX2 and Osx, two transcriptional factors involved in osteoblast differentiation, has been demonstrated in OA [71].

In particular, two subgroups of osteoblasts characterized by a different metabolic status have been described in OA: low OA osteoblasts, which express low levels of prostaglandin E2 (PGE2), IL-6, and OPG, and high RANKL level; high OA osteoblasts, which express high levels of PGE2, IL-6 and OPG, and low RANKL level [72,73]. Low OA subchondral osteoblasts express high levels of ephrin B4 (EphB4) receptor and high levels of membranous RANKL compared to normal and high OA osteoblasts [67,68,74]. The binding of EphB4 receptor to EphB2 inhibits the production of IL-1β, IL-6 and RANKL, but not of OPG [67,68], suggesting a role in abnormal subchondral bone metabolism in OA. Membranous RANKL is enhanced by vitamin D3, PGE2, IL-1β, IL-6, and TNF-α [74], suggesting that low OA osteoblasts play a role in favoring bone resorption, and that high OA osteoblasts are responsible for bone formation.

OA subchondral bone osteoblasts induce a defective mineralization and abnormal organization of the extracellular matrix [75], which is characterized by an altered ratio of α1 and α2 chains of type I collagen, with a prevalence of the α1 chain. OA osteoblasts play also a role in the degradation of articular cartilage by producing hepatocyte growth factor (HGF). HGF stimulates the expression of TGF-β, which induces DKK-2 expression, and inhibits osteoblast response to BMP-2 [76]. As demonstrated in vitro, osteoblasts may also be responsible for abnormal expression of A disintegrin and metalloproteinase with thrombospondin motifs (ADAMTS), MMPs (in particular MMP-13), and cathepsin K involved in the enzymatic degradation of cartilage in OA [77,78,79,80].

Moreover, a role for systemic and local factors has been described in OA osteoblasts. In OA subchondral osteoblasts, high alkaline phosphatase activity and high osteocalcin levels have been described [81,82,83,84]. In OA, osteoblasts express high levels of leptin [85], which in turn induce the production of alkaline phosphatase and osteocalcin [86]. Moreover, vitamin D supplementation is involved in the production of osteocalcin in osteoarthritic osteoblasts [84,87,88]. As demonstrated in vitro, the response of osteoblasts to vitamin D3 stimulation appears to be proportional to the degree of joint damage [84].

OA bone is characterized by alterations of vascularization, which may be responsible for hypoxia. Hypoxia may modify osteoblast phenotype by altering the expression of genes involved in bone matrix regulation, bone remodeling, and bone vasculature [89], favoring the production of leptin, PGE2, cyclooxygenase 2, angiopoietin-like 4, insulin-like growth factor (IGF) binding protein 1 and type II collagen α1 chain [89,90]. Osteoblasts are also able to express VEGF, which is probably involved in the pathogenic mechanisms responsible for the typical bone alterations of OA. VEGF expression has been found higher in primary human OA osteoblast cultures than in normal and osteoporotic osteoblasts, both under basal conditions than in the presence of vitamin D3. Moreover, vitamin D3 significantly enhanced VEGF expression in normal and pathological osteoblasts, suggesting the key role of vitamin D3 supplementation in metabolic bone diseases [91].

## 6. Osteoblast Role in Osteoporosis

Osteoporosis is a disorder characterized by an imbalance of bone turnover, resulting in a reduced bone mineral density, alteration of bone microarchitecture and an increased risk of fracture [92].

A pathological imbalance between bone resorption and bone formation, with a prevalent bone resorption has been seen in osteoporosis. Thus, osteoblast and osteoclast play a key role in the pathogenesis of osteoporosis. On one hand, in postmenopausal osteoporosis the reduced bone mineral density is due to the increased bone resorbing activity of osteoclasts induced by estrogen deficiency. In fact, estrogens are involved in favoring osteoblast activity and inhibiting osteoclastogenesis by inducing OPG production and inhibiting several osteoclastogenesis factors, such as IL-1, IL-6, IL-7, and TNF-α [93,94]. On the other hand, an insufficient osteoblast activity is involved in senile osteoporosis [95].

Local and systemic factors can play a role in the activity of osteoblasts and osteoclasts leading to an imbalance in resorptive activity [96]. Nevertheless, an increased tyrosine phosphorylation of IGF-1 receptor has been seen in osteoblast cultures obtained from patients affected by osteoporosis [97], suggesting that a constitutive alteration of osteoblast activity is probably involved in the pathogenesis of osteoporosis. Recently, a role for microRNAs (miRNAs), such as miR-221, has been described in osteoporosis. In fact, miR-221 down-regulation has been demonstrated in osteoporosis. Considering miR-221 inhibits expression of RUNX2, a transcriptional factor for osteoblast differentiation, it is conceivable that osteoblast differentiation may be dysregulated in osteoporosis [98].

Bisphosphonates, which are commonly used in clinical practice for the treatment of osteoporosis, exert different cellular biochemical effects depending on dosage and their positive effect on bone mineral density could be related both to an increased osteoblast proliferation, and to inhibition of apoptosis of bone forming cells [4,99].

The observation that adipocytes and osteoblasts originate from the same precursors and that increased adipose tissue in bone marrow is associated with decreased bone tissue in osteoporotic patients, suggests a role for adipogenic process in bone loss [100]. Moreover, a reduced differentiation of mesenchimal stem cells in osteogenic cells has been demonstrated in osteoporosis with a compensatory increment of adipocyte differentiation [101,102,103].

Murine studies have suggested a role for OPG deficiency and upregulation of RANKL in increasing bone resorption [104,105,106].

One of most common causes of osteoporosis is chronic glucocorticoid therapy. On one hand, chronic glucocorticoid treatment at high dosage is responsible for both inhibiting proliferation and activity of osteoblasts, and favoring apoptosis of osteoblasts and osteocytes [107]. Glucocorticoids probably inhibit bone formation via suppression of Wnt signaling, as suggested by an in vitro study which has observed high levels of DKK-1 mRNA in cultured human osteoblasts treated with dexamethasone [108]. On the other hand, chronic glucocorticoid treatment at high dosage induces osteoclast activation via stimulating RANKL expression and inhibiting OPG production [107].

## 7. Effects of Immunosuppressive and Biological Therapies on Osteoblast

Inflammatory cytokines play a key role in inducing both generalized and local bone loss, resulting in osteoporosis and joint erosions, respectively. In this regard, in inflammatory diseases, such as RA and SpA, neutralization of these cytokines may also exert useful effects on bone cells.

By studying biochemical markers of bone metabolism, anti-TNF-α therapy has shown inhibitory effects on osteoclast activity, while effects on osteoblasts activity are limited [109,110,111,112,113,114].

An increased osteoblast proliferation has been seen in RA primary osteoblast cell cultures after exposition to the anti-TNF-α monoclonal antibody infliximab [43]. On the contrary, proliferation and metabolic activity of RA osteoblasts has been inhibited by methotexate [43]. Nevertheless, both methotrexate and infliximab have been correlated to a reduced osteoblast production of IL-6, which is involved in osteoclastogenesis [43]. Moreover, IL-6 down-regulation has also been demonstrated in osteoblast cultures exposed to sera of RA patients treated with infliximab [42].

With regard to SpA, Kwon et al. [115] have found a decrease in the number of osteoblast-lineage cells from peripheral blood obtained from AS patients after treatment with infliximab, suggesting a possible useful effect against osteoblastogenesis at the site of inflammation and bone apposition.

## 8. Concluding Remarks

There is accumulating evidence on the role of osteoblasts in RA, SpA, OA and osteoporosis. In fact, osteoblast differentiation, growth and activity are often dysregulated in these diseases. A full understanding of the pathogenic mechanisms involved in osteoblast dysregulation could lead to the development of new therapeutic strategies in these diseases.
